# Ground Rules for Preschooler Exposure to the Digital Environment: A Review of Studies

**DOI:** 10.11621/pir.2023.0403

**Published:** 2023-12-01

**Authors:** Elena I. Nikolaeva, Inna A. Kalabina, Tatyana K. Progackaya, Evgeniya V. Ivanova

**Affiliations:** a Herzen State Pedagogical University of Russia, St. Petersburg, Russia; b Skysmart online school

**Keywords:** digital environment, digital devices, parents, preschool children, guidelines, ground rules

## Abstract

**Background:**

The range of digital technologies that children use from an early age has expanded significantly. Most studies demonstrate that preschoolers now spend substantially longer on digital devices and start using them at a younger age. Finding a solution for this challenge has research merits and relevance, as the data on benefits and harm of early preschoolers’ exposure to digital devices is contradictory. This poses a need to determine theoretically sound and practically validated criteria that could guide the duration and quality of children’s exposure to the digital environment.

**Objective:**

To review studies that contain recommendations on preschoolers’ exposure to the digital environment, namely, exposure limits and evidence to justify the limitation of preschoolers’ time on digital media.

**Design:**

The analysis starts by identifying theoretical foundations that researchers use in their studies of children’s behavior in the digital environment. This is followed by an overview of 40 studies that include research papers, official reports, and methodological recommendations made by healthcare and governmental organizations.

**Results:**

The review identified the following ground rules for children’s exposure to the digital environment: to provide for child’s interaction with a digital device, to use educational applications that will develop skills appropriate to the child’s age, to ensure mandatory supervision of children’s engagement by an adult who limits the exposure according to child’s age-related capabilities and creates conditions for active exploration of the real rather than a virtual world. Children’s cognitive development suffers the most from passive intake of digital content.

**Conclusion:**

The data herein can help to develop strategies to promote healthy and educational engagement of children with digital devices and media; however, the review highlights the insufficiency of psychophysiological research that would make it possible to practically validate the recommendations on the duration of preschoolers’ exposure to the digital environment.

## Introduction

Today’s preschoolers are the first generation to grow and develop fully in the new digital environment and even to be known as digital natives (Sharkins, 2016). Whereas pre-pandemic, it was thought that even primary school children should not be exposed to the digital environment for longer than 20 minutes a day, during the pandemic the duration of digital technology use by children increased significantly (Limone & Toto, 2021; Nikolaeva et al., 2021; Uġraş et al., 2023; Zhang et al., 2022). Today’s children are often introduced to an information-dense digital environment before they turn one year old. They have daily access to new opportunities that are not available in the real world around them (Elias & Sulkin, 2017; Ewin et al., 2021; Griffith et al., 2020). Children in Russia were found to receive a personal digital device (smartphone, tablet, smart watch, etc.) when they are 3 to 6 years old (the data range is 42% to 68%) (Kalabina & Progackaya, 2021; Korotkova et al., 2018).

According to parents in Russia surveyed by Nikolaeva and Isachenkova (2022), 10.2% of children under the age of four have their own digital device (“gadget”). In this group, 1% of the children were still under the age of two. Other research shows that preschoolers have good technical skills to confidently use digital devices, especially through touchscreens (Chaudron et al., 2018; Kalabina & Progackaya, 2021; Papadakis et al., 2021; Veraksa et al., 2020). From an early age, children are surrounded by various electronic devices and mobile information tools. Their impact on children’s physical, mental, and socio-emotional development is poorly understood (Blackwell et al., 2014; Kılıçer & Çoklar, 2015; Plowman et al., 2010). There are even fewer studies of very young children (Elias & Sulkin, 2017; Twenge, 2019).

### Theoretical Basis for Assessing Time Limits on Preschoolers’ Exposure to Digital Media

Prior to assessing the scientific foundation, we would like to emphasize that our definition of “screen time” covers the time children spend on a particular gadget or at the computer plus the time children stay around a working TV. This enhanced definition reduces the novelty of the problem, yet makes it all the more relevant, since some parents leave their children next to a working TV all day long to keep them away from gadgets (Kirkorian et al., 2016, 2018).

### Screen Time and Television

Electromagnetic waves emitted by TVs, together with the sense of security children feel being around adults, are known to produce a reflex, such that a television relieves anxiety and calms children down. This contributes to a lifelong dependence on keeping the television on (Kubey & Csikszentmihalyi, 1990).

Television has been present in children’s lives for quite some time, and there have been longitudinal studies of screen time. Some studies showed that when a child under one year of age watched adult TV programs, the quality of this child’s executive functions at age 4 was impaired and his/her linguistic abilities in elementary school suffered (Barr, 2019; Scarf & Hinten, 2018). Moreover, every extra hour of TV screen time before one year of age was shown to weaken the child’s attention by 28% at age 7 (Christakis, 2004). And if the TV is on while the child and an adult are playing, the adult is less cognizant of the child’s needs and does not speak to the child or respond to his/her questions quite as often. This generally degrades the quality of their communication (Hanson et al., 2021).

### Critical Periods of Early Ontogenesis

One of the most important scientific concepts providing the foundation for an overwhelming number of researchers is the theory of critical periods of early ontogenesis. The theory describes special periods of brain structure plasticity at certain stages in child development (Carson et al., 2015).

Imprinting is one of the first postnatal periods. At this time children capture their parent’s image as an ideal benchmark. That explains why researchers are concerned about parental behavior and their screen time, since children have been found to copy them once they become adults (Corkin, 2021). The other most important critical period is that of speech development, which terminates by the end of the preschool age. It has been repeatedly shown that normative speech development begins with the use of personal speech that accompanies a child’s independent play. In one study, five-year-old children were first asked to build a tower with physical blocks, and then to do the same using a tablet. The study registered a significant reduction in speech activity when the task was performed on the gadget (Bochicchio et al., 2022).

### J. Piaget’s Theory of Children’s Cognitive Development

The concept of critical periods to a great extent ties in with the theory of J. Piaget (Piaget, 1965), which describes preschoolers’ intelligence development. The main theoretical construct is that sensorimotor coupling acts as an equivalent of notions used by adults. The coupling is achieved when the child explores real world objects and engages with them. According to Piaget, prior to facing a symbolic representation of an object in a picture or in a verbal description, the child must get acquainted with it in the real world, feel it, lick it, and otherwise try to interact with it. Only this sequence will shape object’s mental schema in the child’s mind and enable him or her to cognize the object’s symbolic form. Figuratively speaking, the child must first see a chicken, and then listen to “The Speckled Chicken” fairy tale. Disregarding this process was found to impair the formation of cognitive functions and to result in an inability to discriminate the trustworthiness of sources (Richert et al., 2010).

Piaget’s proposition initiated a great number of works confirming its relevance to the use of gadgets with content inappropriate to a child’s age. We have already mentioned that watching adult programs on TV by children under 2 years reduces their cognitive abilities later on. First of all, a child under 2 years of age has a limited understanding of the content on 2D screens (Radesky et al., 2016). The transition to 3D contexts develops slowly during early childhood. Consequently, children of this age cannot relate a complex video image — including complex speech expressions, often unknown to the child — to reality (Ziemer & Snyder, 2016).

One part of Piaget’s theory has a direct match with all the provisions of child learning and cognitive development theories (Liberman,2021). It states that appropriation of information requires the learner to stay active. It also warns about negative consequences that passive information acquisition will have primarily on cognitive control, which is the most important parameter responsible for a child’s behavioral changes and metacognitive functions (Marulis & Nelson, 2021).

### The Role of Motor Activity in Learning

Motor activity is an essential component of child development and learning. The validity of a baby’s picture of the real world is determined by the development of his or her vestibular system (Kim, Avraham, & Ivry, 2021). The accuracy of the picture that the brain creates by analyzing information from receptors is achieved via image corrections based on the head-to-body position. To give three-dimensionality to a flat picture of the surrounding environment presented on the retina as the brain processes information, children must crawl, run or walk to the object many times from different angles, holding their heads in different positions. For that reason unlike all other sensory systems, the vestibular system does not have a simple analyzer in the brain, but rather embraces all brain structures like an octopus. That makes it possible to ensure sensorimotor integration. The less children move, the less accurate is their picture of the outside world (Noel & Angelaki, 2022), the poorer are their metacognitive abilities — that is, the ability to monitor their own cognition (Alvarez-Bueno et al., 2017; Baliram & Ellis, 2019; Escolano-Perez, Herrero-Nivela & Anguera, 2019; Chen & McDunn, 2022).

When preschoolers are given a gadget — instead of less colorful (or sometimes just black and white) book images, where they are required to make an effort to either recognize numbers or letters or even to read — they have sensory experiences which may eventually substitute for other forms and ways of obtaining sensory information. This potentially poses a risk to the child’s normal psychophysical development (Tsai et al., 2017; Woodward, et al., 2016). The most alarming evidence is that vivid images produced by gadgets and viewed by the child before he or she actively engages with the real world (i.e., before the age of one year) make the child feel “bored” in non-virtual reality, as its stimulation is less intensive. Later this was found to predetermine the vector of the child’s cognitive development to a significant degree (Wolf, 2021). The intensive and uncontrolled use of digital technologies at an early age detaches children from true sources of development and poses a serious risk (Smirnova et al., 2018).

Encountering an image on television for the first time may positively affect some cognitive processes and accelerate their progress (Scarf & Hinten, 2018). However, it was found to have a negative effect later on, as children preferred passive actions with gadgets to active learning that would require resolving complex cognitive tasks (Madigan et al., 2020). The earlier a child encounters this attractive tool, the more likely he or she was found to choose passive perception over active learning (Kerai et al., 2022).

For that matter, learning applications developed for touchscreen devices promote children’s active cognition and, as a consequence, improve their working memory, if compared to passively watched TV (Kirkorian et al., 2016; Papadakis, 2023; Papadakis et al., 2021; Vaiopoulou et al., 2022). These applications are developed on the same theory discussed above and factor in the specifics of sensorimotor integration when children perceive information.

### Sensorimotor Integration

The concept of sensorimotor integration requires that children under 4 years of age be presented with information on television at a slow pace and that images be maximally realistic and recognizable (Lillard, et al. 2015). But this does not engage the child’s taste, vestibular, and olfactory sensations. Therefore, screen time should be limited to ensure that the child’s real world activities are not hindered (Suggate & Martzog, 2020). In this regard, some authors emphasize that parents have time limits for interaction with the child, and therefore the more screen time children have, the less active they are in the real world (Scarf & Hinten, 2018).

### Epigenetic Influences

One more aspect is related to the epigenetic impact the early life environment has on the child’s subsequent development. It has been demonstrated that certain upbringing conditions will contribute to changes in gene activity that in turn change the way human body functions (McLaughlin, Weissman, & Bitrán, 2019). One of the most important influences is attributed to stresses experienced in childhood. Stresses suppress genes that regulate responses to stress (Meaney & Szyf, 2005). Quite often parents give gadgets to their children to calm them down in tense situations, in which the parents are afraid of a public failure to pacify them by other means (Shin, 2021). This problematic solution starts a vicious cycle, allowing the children to use outside observers as leverage and to force their parents to give them the gadget by throwing a tantrum. Instead of resolving the conflict, this causes regular stress for both parents and children. Stress-released cortisol can be removed from the bloodstream only through motor activity (Kim, Avraham, & Ivry, 2021), which in this case is substituted by a gadget.

### Socioemotional Development

Finally, children’s socioemotional development is the most important theoretical aspect addressed in many works. Preschool age is a critical period for socioemotional development (Desmarais et al., 2021; Wan, 2021). Many Russian parents show their preschoolers cartoons that were made for children in the USSR. These TV cartoons often have a very strong moral aspect. Parents tend to think this will contribute to the development of child’s moral standards. A study by Mares et al (2018) in the United States examined the prosocial behavior of children who were 3 to 5 years old. In the research, 107 children watched cartoons that presented moral behaviors. It turned out that the preschoolers did not understand the content well enough and in the subsequent behavioral tests they did not demonstrate the behaviors promoted in the cartoons.

Many studies are contradictory (Cajochen et al., 2011; Clowes, 2018; Coiro, 2020), which highlights the need to develop evidence-based recommendations that would provide ground rules for preschoolers’ exposure to the digital environment. These recommendations should correspond not only to researchers’ theoretical assumptions, but also to the realistic capacity of families with children. All of the above has determined the purpose of this article: to review papers that — at varying depth — provide evidence for ground rules for preschoolers’ exposure to the digital world.

## Methods

Given the conflicting evidence on the benefits and harm of preschoolers’ experiences with digital devices and media, this article analyzes research, official reports, and methodological recommendations made by healthcare and governmental organizations that contain data and guidelines on preschool children’s exposure to digital media. The literature search for this review was done via Google Scholar, PubMed, Research Gate, and Web of Science. Search keywords: digital environment; digital devices; digital media; early childhood smart devices; impacts of screen time; screen exposure; screen time; parental mediation; preschool children; preschooler cognitive development; preschooler socioemotional development. The review includes studies that: 1) contained proposals or requirements that could form a regulatory framework, describing the rules of digital exposure for children aged 4–7 years; 2) contain justifications for limiting the time preschoolers spend on digital media. A total of 40 publications were reviewed: 11 publications referred to preschoolers’ time in the digital environment and 29 publications had research data on how the digital environment impacts preschoolers. These studies in varying degrees justify recommendations and restrictions on preschoolers’ time in the digital environment. Publications that only cited limits on preschoolers’ exposure to the digital environment and findings of earlier studies were not included in our review.

## Results

### Guidelines for How Long Preschoolers May Spend on Digital Devices

Although digital technologies are increasingly important in our lives, some researchers claim that they harm children’s health (Anderson & Rainie, 2018). Perhaps most telling was a cognitive ability study of video gamers who began gaming as children in the 1980s and continue to play the games as adults. Excessive gaming time definitely resulted in attention deficit, social communication difficulties, and a higher risk of obesity. But some gamers were found to have cognitive advantages compared to an average subject who does not play computer games. The gamers were highly adapted to processing various types of visual information, had better spatial visualization and shorter response time to external stimulus, and were able to rotate objects in their heads. It was demonstrated that video games limited to 1 hour per day for approximately 4 days a week in 6 months improved the subjects’ visual vigilance (the ability to discern and process visual information), spatial attention, and multitasking (Green & Bavelier, 2003).

In response to this challenge, a number of organizations and researchers have developed specific recommendations on the duration of and conditions for children’s use of digital devices. Of the publications we analyzed, 12 provide direct recommendations about how long children may use digital devices and/or screen time. These recommendations are not quotes from other sources. Table 1 presents the data in the publications’ chronological order.

**Table 1:** Guidelines on how long preschoolers may spend on digital devices

Source	Requirements of digital exposure for children	Type of publication
Australian Department of Health, 2012	Children under the age of 2 should not watch television or use any digital devices.	Report
National Association for the Education of Young Children & Fred Rogers Center for Early Learning Children’s Media at Saint Vincent College, USA, 2012	Limit any use of technology and interactive media in programs for children younger than 2 years. Prohibit the passive use of TV, videos, DVDs, and other non-interactive technologies and media in early childhood programs for children younger than 2 years, and discourage passive and non-interactive uses with children ages 2 through 5.	Position statement
Hill et al., 2016	Avoid introducing children younger than 18–24 months to digital media (except for video chats). For children aged 2–5 years, screen time should be limited to 1 hour per day. The guidelines recommend ensuring high quality content and parent–child media sharing.	Policy statement
Canadian Paediatric Society Digital Health Task Force on Okanagan, 2017	Screen time for children under 2 years of age is not recommended, whereas for children aged 2 to 5 years the limit is 1 hour per day and no screen time 1 hour before bedtime.	Position statement
Early Childhood Australia, 2018	Screens should be used for short time periods with regular breaks; screen time should not be a substitute for physical activity and digital devices and screens should not be used before bedtime.	Report
World Health Organization, 2019	For children under 1 year of age, digital media and TV viewing are not recommended. For children aged 2 to 4 years, sedentary screen time should not exceed 1 hour.	Report
Soldatova & Vishneva, 2019	Preschoolers aged 5–6 years ideally shall not use the Internet and digital devices for longer than 1 hour per day.	Research paper
Royal College of Paediatrics and Child Health, United Kingdom, 2019	Families should negotiate screen time limits with their children based upon the needs of an individual child, the ways in which screens are used, and the degree to which use of screens appears to displace (or not) physical and social activities and sleep.	Guide
Sanitary Regulations and Norms, Russian Federation, SanPiN 2.4.3648-20, 2020	Continuous screen use should not exceed 5 to 7 minutes for children aged 5–7 years and 10 minutes for students in grades 1 through 4 and shall alternate with mandatory eye exercises and physical education breaks. Mobile digital devices are not used for educational purposes. Electronic educational devices are not used for children under 5 years of age.	Decree
Hygienic norms and special requirements for the device, content and modes of work in the conditions of digital educational environment in the field of general education. Guidelines, Ministry of Health of the Russian Federation, 2020	The total recommended daily time spent on digital devices and e-learning tools for children aged 6–7 years is 80 minutes. It increases to 90 minutes in grade 3 (starting 9 years of age).	Guide
Pivovarova et al., 2021	No use of tablets or mobile phones by children under 2 years; screen time for children aged 2 to 5 years should not exceed 1 hour per day; avoid using gadgets 1 hour before bedtime; no background TV.	Research paper

These guidelines make a significant contribution to shaping the environment that will promote preschooler development, especially with regard to digital exposure. However, the evidence for the guidelines is not always clear. More evidence is needed to separate the impact of screen time from other factors influencing children’s health (Ashton & Beattie, 2019).

### Justification for Limiting Preschooler Exposure to the Digital Environment

Some publications contain data on how various aspects of children’s physical health and development are impacted by excessive exposure to a digital environment. Reduced motor activity of children nowadays, as it is replaced by digital device engagement, has been found to result in degradation of children’s physical aptitude, poor development of fine motor skills (Binnur 2015), overweight and poor health (Anderson, 2008; Marsh et al., 2013; McVeigh et al., 2016).

Children were found to be less exposed to sunshine because they spend more time on digital devices and stay indoors (Dresp-Langley, 2020). Continuous screen watching at a close range has been associated with vision problems in adolescents (Kim et al., 2016). The bright light from digital screens and digital content was found to excite the child and impact the falling asleep stage and sleep quality (Carter et al., 2016; Cheung et al., 2017; Lin et al., 2021). Though some studies found no correlation between sleep quality and a child’s tablet or mobile phone use, they identified a risk of sleep disorders as TV viewing time increases (Zhu et al., 2020).

Anxiety and depression triggered by excessive use of digital devices has been shown in studies of adolescents and is primarily associated with social media use. On the other hand, social media can be a good tool for children to develop social connections, especially for children with disabilities (Perezhogin, 2022). There were no studies of depression in preschoolers caused by digital device use. Moreover, many preschoolers associate the use of digital devices with positive experiences (Kalabina & Progackaya, 2021). Children feel joy when they succeed in digital games and applications (Warburton & Highfield, 2017).

The negative impact of digital devices has also been linked to behavioral problems in children. Lin and colleagues (2020) demonstrated that children aged 18 months to 3 years who use touchscreens experienced emotional and behavioral problems. A four-year longitudinal study tested the hypothesis that use of digital devices such as smartphones and tablets by 4-year-olds is associated with dysregulation symptoms and lower academic achievement at the age of 6–8. Another study found that a child’s screen time at age 4 is directly, positively, and significantly associated with dysregulation and negatively associated with math and literacy scores at age 8 (Cerniglia et al., 2020).

Positive outcomes were found by some researchers who demonstrated that early digital experiences of young children are beneficial for their cognitive development. However, these experiences may potentially negatively affect social and emotional development caused by a delayed development of age-appropriate social interaction skills (Cabré-Riera et al., 2019; Pecherskaya et al., 2013). Developing ways in which large screens in kindergartens are shared to create a single product can promote children’s critical thinking and prosocial behavior (Sundararajan, Adesope, & Cavagnetto, 2018).

Positive effects of digital device use on regulatory functions, auditory working memory, cognitive flexibility, and inhibitory control have been confirmed in preschoolers who use digital devices once a week, compared to children who use them 3–4 times a week (Veraksa et al., 2022). Also, the appropriate use of digital technology can stimulate creative activities and promote creative abilities in young children (Fielding & Murcia, 2022).

Authors disagree on the effects of video games on cognitive abilities. The impact depends on gaming intensity and type as well as on the gamer’s personal characteristics (Vedechkina & Borgonovi, 2021; Walsh et al., 2020).

A study that explored the association between the age when children start using gadgets and the cumulative effects of digital exposure through the initial 2 years of life on the one hand, and cognitive development at age 4 on the other, found that cognitive development at age 2 positively correlated with a later age of digital device use and with a shorter intense exposure to screen media (Supanitayanon et al., 2020).

It is important to distinguish between active and passive screen time. Passive screen time of 5-year-olds was found to correlate negatively with their math and science achievements, executive function, and social skills. In a study of Chinese children, active time in front of a screen correlated positively with the children’s language skills and knowledge of science (Hu et al., 2020).

A longitudinal study that identified how passive viewing and active use of digital resources correlate with preschoolers’ executive function and psychosocial development concluded that limiting the use of electronic applications to 30 minutes or less per day and limited multimedia app viewing may positively correlate with preschoolers’ cognitive and psychosocial development (McNeill et al., 2019).

Longer screen use (hours per day/week) was negatively associated with children’s language skills, whereas higher quality use of digital devices (e.g., using educational programs or watching together with adults) was positively associated with children’s language skills (Madigan et al., 2020). A longitudinal study in families of children in Taiwan showed an association between parental involvement, children’s screen time, and their social competence. In children aged 3 to 5 years, parental mediation correlated positively with the children’s social competence, whereas time spent by children in front of a screen correlated negatively with their social competence (Ma et al., 2022). These studies support pediatric recommendations to limit children’s screen time, to choose high-quality programs, and to assure joint child–adult use of digital devices.

An association was identified by Hutton and colleagues (2022) between longer digital media use and less cortical thickness and sulcus depth in brain regions that are responsible for primary visual processing and for higher-order functions such as top-down attention, complex memory encoding, letter recognition, and social cognition. These outcomes are consistent with the findings by those researchers’ earlier study of adolescents and suggest that differences in the cortical structure associated with screen use may become apparent in early childhood (Hutton et al., 2022). Digital device use for longer than the times recommended by the American Academy of Pediatrics was found to be associated with lower scores of microstructural organization and myelination of brain white matter tracts that support speech and literacy development (Hutton al., 2020).

## Discussion

Most of the featured studies are based on the evidence collected through sociological or psychological surveys of educators, parents, and teachers. The psycho-physiological approach to guidelines on the exposure to the digital environment is often neglected, as it is difficult to do such research with preschoolers. Most papers analyze “screen time,” i.e., the time that the child passively perceives some content, while in fact many children actively engage with characters performing on the screen. The real screen time of preschoolers has been found to exceed recommended limits (Hu et al., 2020; Kalabina & Progackaya, 2021; Kornienko et al., 2022; Nikolaeva & Isachenkova, 2022; Soldatova & Vishneva, 2019). Surveys of preschoolers’ parents showed that the time parents spent on digital devices closely correlated with their child’s screen time (Lauricella et al., 2015; Lin et al., 2021).

Research shows both positive and negative effects of screen time and digital technology. It also demonstrates an association with the duration and frequency of device use. Quite often researchers do not include the positive impact of early age digital literacy in their outcome measures (Ashton & Beattie, 2019), though digital competence is seen as a key skill in the world today and is necessary for lifelong learning (Cortesi et al., 2020; Kalabina & Progackaya, 2022). An important recommendation refers to the quality of and context wherein the content is watched and whether it is discussed with an adult.

Key recommendations to parents and teachers include the reduction of total screen time for preschoolers, the use of quality content, and the adult–child joint use of digital technologies (Hill, 2016; Royal College of Paediatrics and Child Health, 2019). Despite the importance of a family’s social profile and its geographical location, the recommendations are universal as they are based on the child age-related profile. However, the region of domicile (e.g., its climatic, cultural, economic, and other parameters) has an established relationship with preschoolers’ screen time (Kornienko et al., 2022). Three studies indirectly support the guidelines on digital device use and screen time (Madigan et al., 2020; McNeil et al., 2019; Hutton, 2020).

Research into and discussions of the challenge with an exclusive focus on screen time seem inadequate. Today’s preschoolers engage with voice assistants, smart speakers, and interactive toys connected to the internet. These tools are also part of the digital environment. The general challenge is the fast pace of technological changes, while researchers are unable to keep up in their study of how technologies affect children (Komarova, 2022). The impact of virtual reality and immersive technologies on children deserves a separate discussion (Bailey & Bailenson, 2017), but there is not yet much data specifically on preschoolers. Compared to their older peers, young children are more likely to perceive any digital content as real, and this may influence their behavior (Richert et al. 2011).

## Conclusion

Rapid digital transformation and digital technologies that penetrate all domains of children’s lives do not allow the construction of defensive strategies if they focus only on limiting the use of technology. Focusing on the influence of screen time and limiting exposure to it do not harness the digital world’s benefits to stimulate preschooler development. On the whole, the data presented in the studies we reviewed enable us to describe some specifics about how cognitive abilities and their psychophysiological mechanisms develop in preschool children who have different experiences of digital socialization. However, the data is incomplete and contradictory. Nevertheless, theoretical concepts regarding critical periods in early ontogenesis, sensorimotor integration, and motor activity in the development of cognition, allow us to identify some ground rules of children’s exposure to the digital environment, namely: to stay active while engaging with a digital device, to use educational applications that will develop skills appropriate to the child’s age, to ensure mandatory supervision of the child’s engagement by an adult who limits the exposure according to child’s age-related capabilities and creates conditions for active exploration of a real rather than virtual world. Children’s cognitive development suffers the most from a passive intake of digital content.

## Limitations

The study scope is limited as it describes and reviews publicly available research and full-text guidelines.

## References

[ref1] Alvarez-Bueno, C., Pesce, C., Cavero-Redondo, I., Sanchez-Lopez, M., Martínez-Hortelano, J.A., & Martinez-Vizcaino, V. (2017). The effect of physical activity interventions on children’s cognition and metacognition: A systematic review and meta-analysis. Journal of the American Academy of Child & Adolescent Psychiatry, 56(9), 729–738. 10.1016/j.jaac.2017.06.012 28838577

[ref2] Anderson, J., & Rainie, L. (2018). The future of well-being in a tech-saturated world. Retrieved from https://www.pewresearch.org/internet/2018/04/17/the-future-of-well-being-in-a-tech-saturated-world/

[ref3] Anderson, S.E., Economos, C.D., & Must, A. (2008). Active play and screen time in US children aged 4 to 11 years in relation to sociodemographic and weight status characteristics: A nationally representative cross-sectional analysis. BMC Public Health, 8, 366. 10.1186/1471-2458-8-366 18945351 PMC2605460

[ref4] Ashton, J.J., & Beattie, R.M. (2019). Screen time in children and adolescents: Is there evidence to guide parents and policy? The Lancet Child & Adolescent Health, 3(5), 292–294. 10.1016/s2352-4642(19)30062-8 30853301

[ref5] Australian Department of Health. (2012). Inactivity and screen time. Retrieved from http://www.health.gov.au/internet/publications/publishing.nsf/Content/gug-indig-hb~inactivity

[ref6] Bailey, J.O., & Bailenson, J.N. (2017). Immersive virtual reality and the developing child. Cognitive Development in Digital Contexts, 181–200. 10.1016/b978-0-12-809481-5.00009-2

[ref7] Baliram, N., & Ellis, A.K. (2019). The impact of metacognitive practice and teacher feedback on academic achievement in mathematics. School Science and Mathematics, 119(2), 94–104. 10.1111/ssm.12317

[ref8] Barr, R. (2019). Growing up in the digital age: Early learning and family media ecology. Current Directions in Psychological Science, 28(4), 341–346. 10.1177/0963721419838245 31423053 PMC6697422

[ref9] Binnur, J.I. (2015). How does technology affect language learning process at an early age? ScienceDirect. Procedia–Social and Behavioral Sciences, 199, 311–316. DOI:10.1016/j.sbspro.2015.07.552

[ref10] Blackwell, C.K., Lauricella, A.R., & Wartella, E. (2014). Factors influencing digital technology use in early childhood education. Computers & Education, 77, 82–90. 10.1016/j.compedu.2014.04.013

[ref11] Bochicchio, V., Keith, K., Montero, I., Scandurra, C., & Winsler, A. (2022). Digital media inhibit self-regulatory private speech use in preschool children: The “digital bubble effect.” Cognitive Development, 62, 101180. 10.1016/j.cogdev.2022.101180

[ref12] Cabré-Riera, A., Torrent, M., Donaire-Gonzalez, D., Vrijheid, M., Cardis, E., & Guxens, M. (2019). Telecommunication devices use, screen time and sleep in adolescents. Environmental Research, 171, 341–347. 10.1016/j.envres.2018.10.036 30716511

[ref13] Cajochen, C., Frey, S., Anders, D., Späti, J., Bues, M., Pross, A., … & Stefani, O. (2011). Evening exposure to a light-emitting diodes (LED)-backlit computer screen affects circadian physiology and cognitive performance. Journal of Applied Physiology, 110(5), 1432–1438. 10.1152/japplphysiol.00165.2011 21415172

[ref14] Canadian Paediatric Society Digital Health Task Force on Okanagan. (2017). Screen time and young children: Promoting health and development in a digital world. Paediatrics & Child Health, 22(8), 461–477. 10.1093/pch/pxx123 29601064 PMC5823000

[ref15] Carson, V., Kuzik, N., Hunter, S., Wiebe, S.A., Spence, J.C., Friedman, A., … & Hinkley, T. (2015). Systematic review of sedentary behavior and cognitive development in early childhood. Preventive Medicine, 78, 115–122. 10.1016/j.ypmed.2015.07.016 26212631

[ref16] Carter, B., Rees, P., Hale, L., Bhattacharjee, D., & Paradkar, M.S. (2016). Association between portable screen-based media device access or use and sleep outcomes. JAMA Pediatrics, 170(12), 1202–1208. 10.1001/jamapediatrics.2016.2341 27802500 PMC5380441

[ref17] Cerniglia, L., Cimino, S., & Ammaniti, M. (2020). What are the effects of screen time on emotion regulation and academic achievements? A three-wave longitudinal study on children from 4 to 8 years of age. Journal of Early Childhood Research, 19(2), 145–160. 10.1177/1476718x20969846

[ref18] Chaudron, S., Di Giota, R., & Gemo M. (2018). Young children (0–8) and digital technology, a qualitative study across Europe. Publications Office of the European Union. Retrieved from https://publications.jrc.ec.europa.eu/repository/handle/JRC110359

[ref19] Chen, S., McDunn, B.A. (2022). Metacognition: History, measurements, and the role in early childhood development and education. Learning and Motivation, 78, 101786. 10.1016/j.lmot.2022.101786

[ref20] Cheung, C.H., Bedford, R., Saez De Urabain, I.R., Karmiloff-Smith, A., & Smith, T.J. (2017). Daily touchscreen use in infants and toddlers is associated with reduced sleep and delayed sleep onset. Scientific Reports, 7(1). 10.1038/srep46104 PMC5390665 28406474

[ref21] Christakis, D.A., Zimmerman, F.J., DiGiuseppe, D.L., & McCarty, C.A. (2004). Early television exposure and subsequent attentional problems in children. Pediatrics, 113(4), 708–713. 10.1542/peds.113.4.708 15060216

[ref22] Clowes, R.W. (2018). Screen reading and the creation of new cognitive ecologies. AI & SOCIETY, 34(4), 705–720. 10.1007/s00146-017-0785-5

[ref23] Coiro, J. (2020). Toward a multifaceted heuristic of digital reading to inform assessment, research, practice, and policy. Reading Research Quarterly, 56(1), 9–31. 10.1002/rrq.302

[ref24] Corkin, M.T., Henderson, A.M.E., Peterson, E.R., Kennedy-Costantini, S., Sharplin, H.S., & Morrison, S. (2021). Associations between technoference, quality of parent–infant interactions, and infants’ vocabulary development. Infant Behavior and Development, 64, 101611. 10.1016/j.infbeh.2021.101611 34303915

[ref25] Cortesi, S., Hasse, A., Lombana-Bermudez, A., Kim, S., & Gasser, U. (2020). Youth and digital citizenship+ (plus): Understanding skills for a digital world. Berkman Klein Center for Internet & Society. 10.2139/ssrn.3557518

[ref26] Decree of the Chief State Sanitary Doctor of the Russian Federation (2020). Sanitarno-epidemiologicheskie trebovaniia k organizatsiiam vospitaniia i obucheniia, otdykha i ozdorovleniia detei i molodezhi [Sanitary-epidemiological requirements for organizations of education, recreation and healthcare of children and youth], SanPiN 2.4.3648-20.

[ref27] Desmarais, E., Brown, K., Campbell, K., French, B.F., Putnam, S.P., Casalin, S., … & Gartstein, M.A. (2021). Links between television exposure and toddler dysregulation: Does culture matter? Infant Behavior and Development, 63, 101557. 10.1016/j.infbeh.2021.101557 33878597

[ref28] Dresp-Langley, B. (2020). Children’s health in the digital age. International Journal of Environmental Research and Public Health, 17(9), 3240. 10.3390/ijerph17093240 32384728 PMC7246471

[ref29] Early Childhood Australia (ECA). (2018). Statement on young children and digital technologies. ECA. 10.23965/ECA.001

[ref30] Elias, N., & Sulkin, I. (2017). YouTube viewers in diapers: An exploration of factors associated with amount of toddlers’ online viewing. Cyberpsychology: Journal of Psychosocial Research on Cyberspace, 11(3), 2. 10.5817/cp2017-3-2

[ref31] Escolan o-Pérez, E., Herrero-Nivela, M.L., & Anguera, M.T. (2019). Preschool metacognitive skill assessment in order to promote an educational sensitive response from mixed-methods approach: Complementarity of data analysis. Frontiers in Psychology, 10(2) 1298–1309. 10.3389/fpsyg.2019.01298 31263438 PMC6585472

[ref32] Ewin, C.A., Reupert, A.E., McLean, L.A., & Ewin, C.J. (2021). The impact of joint media engagement on parent–child interactions: A systematic review. Human Behavior and Emerging Technologies, 3(2), 230–254. 10.1002/hbe2.203

[ref33] Fielding, K., & Murcia, K. (2022). Research linking digital technologies to young children’s creativity: An interpretive framework and systematic review. Issues in Educational Research, 32(1), 105–125. Retrieved from http://www.iier.org.au/iier32/fielding.pdf

[ref34] Green, C.S., & Bavelier, D. (2003). Action video game modifies visual selective attention. Nature, 423(6939), 534–537. 10.1038/nature01647 12774121

[ref35] Griffith, S.F., Hagan, M.B., Heymann, P., Heflin, B.H., & Bagner, D.M. (2020). Apps as learning tools: A systematic review. Pediatrics, 145(1). 10.1542/peds.2019-1579 31871246

[ref36] Hanson, K.G., Lavigne, H.J., Gover, S.G., & Anderson, D.R. (2021). Parent language with toddlers during shared storybook reading compared to coviewing television. Infant Behavior and Development, 65, 101646. 10.1016/j.infbeh.2021.101646 34509712

[ref37] Hill, D., Ameenuddin, N., Reid Chassiakos, Y. (Linda), Cross, C., Hutchinson, J., Levine, A., … & Swanson, W.S. (2016). Media and young minds. Pediatrics, 138(5). 10.1542/peds.2016-2591 27940793

[ref38] Hu, B.Y., Johnson, G.K., Teo, T., & Wu, Z. (2020). Relationship between screen time and Chinese children’s cognitive and social development. Journal of Research in Childhood Education, 34(2), 183–207. 10.1080/02568543.2019.1702600

[ref39] Hutton, J.S., Dudley, J., DeWitt, T., & Horowitz-Kraus, T. (2022). Associations between digital media use and brain surface structural measures in preschool-aged children. Scientific Reports, 12(1). 10.1038/s41598-022-20922-0 PMC9645312 36351968

[ref40] Hutton, J.S., Dudley, J., Horowitz-Kraus, T., DeWitt, T., & Holland, S.K. (2020). Associations between screen-based media use and brain white matter integrity in preschool-aged children. JAMA Pediatrics, 174(1). 10.1001/jamapediatrics.2019.3869 PMC6830442 31682712

[ref41] Kalabina, I.A., & Progackaya, T.K. (2021). Defining digital competence for older preschool children. Psychology in Russia: State of the Art, 14(4), 169–185. 10.11621/pir.2021.0411 36733823 PMC9888038

[ref42] Kalabina, I.A., & Progackaya, T.K. (2022). Formirovanie tsifrovoi kompetentnosti detei starshego doshkol’nogo vozrasta [Formation of digital competence of older preschool children]. Sovremennoe doshkol’noe obrazovanie [Preschool Education Today], 2(16), 58–69. 10.24412/1997-9657-2022-2110-58-69

[ref43] Kerai, S., Almas, A., Guhn, M., Forer, B., & Oberle, E. (2022). Screen time and developmental health: Results from an early childhood study in Canada. BMC Public Health, 22(1), 1–9. 10.1186/s12889-022-12701-3 35168575 PMC8845249

[ref44] Kim, H.E., Avraham, G., & Ivry, R.B. (2021). The psychology of reaching: Action selection, movement implementation, and sensorimotor learning. Annual Review of Psychology, 72(1), 61–95. 10.1146/annurev-psych-010419-051053 PMC8514106 32976728

[ref45] Kim, J., Hwang, Y., Kang, S., Kim, M., Kim, T.-S., Kim, J., … & Park, S.K. (2016). Association between exposure to smartphones and ocular health in adolescents. Ophthalmic Epidemiology, 23(4), 269–276. 10.3109/09286586.2015.1136652 27254040

[ref46] Kirkorian, H.L. (2018). When and how do interactive digital media help children connect what they see on and off the screen? Child Development Perspectives, 12(3), 210–214. 10.1111/cdep.12290

[ref47] Kirkorian, H., Choi, K., & Pempek, T. (2016). Toddlers’ word learning from contingent and noncontingent video on touch screens. Child Development, 87(2) 405–413. 10.1111/CDEV.12508 27018327

[ref48] Kılıçer, K., & Çoklar, A.N. (2015). Examining human value development of children with different habits of internet usage. Hacettepe Üniversitesi Egitim Fakültesi Dergisi [Journal of Hacettepe university faculty of education], 30(1), 163–177.

[ref49] Komarova, I.I. (2022). Detskii sad i riski tsifrovoi transformatsii [Kindergarten and risks of digital transformation]. Sovremennoe doshkol’noe obrazovanie [Preschool Education Today], 2(110), 4–15. 10.24412/1997-9657-2022-2110-4-15

[ref50] Kornienko, D.S., Veraksa, A.N., Chichinina, E.A., Bukhalenkova, D.A., & Chursina, A.V. (2022, February). Ispol’zovanie tsifrovykh ustroistv doshkol’nikami, prozhivaiushchimi v severnom i tsentral’nom regionakh Rossii [The use of digital devices by preschool children residing in the northern and central regions of Russia]. Arktika: gumanitarnye vektory razvitiia [The Arctic: Humanitarian vectors of development], Retrieved from https://elibrary.ru/item.asp?id=48141564

[ref51] Korotkova, A., Verlina, Iu., Tsai, T., Nikitina, S., Sosnovaia, A., & Khokhlova, A. (2018). Deti. Mediapotreblenie. 2017 [Children. Media consumption. 2017]. MOMRI — Modern Media Research Institute.

[ref52] Kubey, R.W., & Csikszentmihalyi, M. (1990). Television and the quality of life: How viewing shapes everyday experience. Lawrence Erlbaum.

[ref53] Lauricella, A.R., Wartella, E., & Rideout, V.J. (2015). Young children’s screen time: The complex role of parent and child factors. Journal of Applied Developmental Psychology, 36, 11–17. 10.1016/j.appdev.2014.12.001

[ref54] Liberman, D.A. (2021) Learning and memory. Cambridge University Press.

[ref55] Lillard, A.S., Li, H., & Boguszewski, K. (2015). Television and children’s executive function. Advances in Child Development and Behavior, 48, 219–248. 10.1016/bs.acdb.2014.11.006 25735946

[ref56] Limone, P., & Toto, G.A. (2021). Psychological and emotional effects of digital technology on children in COVID-19 pandemic. Brain Sciences, 11(9), 1126. 10.3390/brainsci11091126 34573148 PMC8465704

[ref57] Lin, H.-P., Chen, K.-L., Chou, W., Yuan, K.-S., Yen, S.-Y., Chen, Y.-S., & Chow, J.C. (2020). Prolonged touch screen device usage is associated with emotional and behavioral problems, but not language delay, in toddlers. Infant Behavior and Development, 58, 101424. 10.1016/j.infbeh.2020.101424 32120178

[ref58] Lin, P., Kuo, S., Lin, Y., & Chen, S. (2021). The relationship between screen time and sleep quality, psychosocial behavior adaptation in preschool children. New Taipei Journal of Nursing, 23(1), 33–42. 10.6540/NTJN.202103_23(1).0004

[ref59] Ma, S., Li, J., & Chen, E.E. (2022). Does screen media hurt young children’s social development? Longitudinal associations between parental engagement, children’s screen time, and their social competence. Early Education and Development, 1–16. 10.1080/10409289.2022.215140135082478

[ref60] Madigan, S., McArthur, B.A., Anhorn, C., Eirich, R., & Christakis, D.A. (2020). Associations between screen use and child language skills. JAMA Pediatrics, 174(7), 665–675. 10.1001/jamapediatrics.2020.0327 32202633 PMC7091394

[ref61] Madigan, S., Racine, N., & Tough, S. (2020). Prevalence of preschoolers meeting vs exceeding screen time guidelines. JAMA Pediatrics, 174(1) 93–95. 10.1001/JAMAPEDIATRICS.2019.4495 31764965 PMC6902205

[ref62] Mares, M.-L., Bonus, J.A., & Peebles, A. (2018). Love or comprehension? Exploring strategies for children’s prosocial media effects. Communication Research, 49(6) 763–791. 10.1177/0093650218797411

[ref63] Marulis, L.M., & Nelson, L.J. (2021). Metacognitive processes and associations to executive function and motivation during a problem-solving task in 3–5 years olds. Metacognition and Learning, 16(1), 207–231. 10.1007/s11409-020-09244-6

[ref64] Marsh, S., Ni Mhurchu, C., & Maddison, R. (2013). The non-advertising effects of screen-based sedentary activities on acute eating behaviours in children, adolescents, and young adults. A systematic review. Appetite, 71, 259–273. 10.1016/j.appet.2013.08.017 24001394

[ref65] McLaughlin, K.A., Weissman, D., & Bitrán, D. (2019). Childhood adversity and neural development: A systematic review. Annual Review of Developmental Psychology, 1(1), 277–312. 10.1146/annurev-devpsych-121318-084950 PMC7243625 32455344

[ref66] McNeill, J., Howard, S.J., Vella, S.A., & Cliff, D.P. (2019). Longitudinal associations of electronic application use and media program viewing with cognitive and psychosocial development in preschoolers. Academic Pediatrics, 19(5), 520–528. 10.1016/j.acap.2019.02.010 30853576

[ref67] McVeigh, J., Smith, A., Howie, E., & Straker, L. (2016). Trajectories of television watching from childhood to early adulthood and their association with body composition and mental health outcomes in young adults. PLOS ONE, 11(4). 10.1371/journal.pone.0152879 PMC4838324 27097324

[ref68] Meaney, M.J., & Szyf, M. (2005). Environmental programming of stress responses through DNA methylation: Life at the interface between a dynamic environment and a fixed genome. Dialogues in Clinical Neuroscience, 7(2), 103–123. 10.31887/dcns.2005.7.2/mmeaney 16262207 PMC3181727

[ref69] Minzdrav Rossii [Ministry of Health of the Russian Federation] (2020). Gigienicheskie normativy i spetsial’nye trebovaniia k ustroistvu , soderzhaniiu i rezhimam raboty v usloviiakh tsifrovoi obrazovatel’noi sredy v sfere obshchego obrazovaniia. Rukovodstvo [Hygienic norms and special requirements for the device, content, and modes of work in conditions of a digital educational environment in the field of general education. Guidelines].

[ref70] National Association for the Education of Young Children & Fred Rogers Center for Early Learning Children’s Media at Saint Vincent College. (2012). Technology and interactive media as tools in early childhood programs serving children from birth through age 8. Retrieved from http://www.naeyc.org/files/naeyc/file/positions/PS_technology_WEB2.pdf

[ref71] Nikolaeva, E.I, Dunaevskaya, E.B, & Kalabina, I.A. (2021). Factors that impact parental success in supporting children’s distance learning. Society. Integration. Education. Proceedings of the International Scientific Conference, 5, 188–198. 10.17770/sie2021vol5.6185

[ref72] Nikolaeva, E.I., & Isachenkova, M.L. (2022). Osobennosti ispol’zovaniia gadzhetov det’mi do chetyrekh let po dannym ikh roditelei [The use of gadgets by children under four years old: Evidence from their parents]. Kompleksnye issledovaniia detstva [Comprehensive Studies of Childhood], 4(1), 32–53. 10.33910/2687-0223-2022-4-1-32-53

[ref73] Noel, J.-P., & Angelaki, D.E. (2022). Cognitive, systems, and computational neurosciences of the self in motion. Annual Review of Psychology, 73(1), 103–129. 10.1146/annurev-psych-021021-103038 PMC8897977 34546803

[ref74] Papadakis, S. (2023). Choosing the best educational apps for young children: What parents and educators need to know. In I.M.G. Trigueros (Ed.), Desafíos de la inclusión digital: la brecha digital de género y las competencias digitales docentes en el contexto educativo [Challenges of digital inclusion: the digital gender gap and teaching digital competencies in the educational context] (pp. 77–94). Octaedro. https://www.researchgate.net/publication/372241089_Choosing_the_best_educational_apps_for_young_children_What_parents_and_educators_need_to_know

[ref75] Papadakis, S., Alexandraki, F. & Zaranis, N. (2021). Mobile device use among preschool-aged children in Greece. Educ Inf Technol, 6, 1–34. Retrieved from https://link.springer.com/article/10.1007/s10639-021-10718-6 10.1007/s10639-021-10718-6 PMC8406380 34483704

[ref76] Papadakis, S., Kalogianakis, M., Sifaki, E., & Monnier, A. (2021). Editorial: The impact of smart screen technologies and accompanied apps on young children learning and developmental outcomes. Frontiers in Education, 6. 10.3389/feduc.2021.790534

[ref77] Pecherskaya, E.P., Zvonovskiy, V.B., Merkulova, D.Yu., Pleshakov, V.A., Matskevich, M.G., & Sablina, O.I. (2013). Internet i deti: sotsial’noe povedenie molodykh rossiian v internete [Internet and children: Social behavior of young Russians on the Internet]. SamGEU Publishing.

[ref78] Perezhogin L.O. (2022). Vliianie sotsial’nykh setei na psikhologicheskoe zdorov’e detei i podrostkov [The impact of social media on the mental health of children and adolescents]. Psikhicheskoe zdorovie [Mental Health], 17(5), 45–54. 10.25557/2074-014X.2022.05.45-54

[ref79] Piaget, J. (1965). The child’s conception of number. W. W. Norton & Company, Inc.

[ref80] Pivovarova, A.M., Shabelnikova, E.I., & Gorchkhanova, Z.K. (2021). Vliianie tsifrovykh tekhnologii na zdorov’e detei [The impact of digital technologies on children’s health]. Praktika pediatra [Pediatrician’s Practice], 4, 12–20.

[ref81] Plowman, L., McPake, J., & Stephen, C. (2010). The technologisation of childhood? Young children and technology in the home. Children & Society, 24(1), 63–74. 10.1111/j.1099-0860.2008.00180.x

[ref82] Radesky, J.S., & Christakis, D.A. (2016). Increased screen time. Pediatric Clinics of North America, 63(5), 827–839. 10.1016/j.pcl.2016.06.006 27565361

[ref83] Richert, R.A., Robb, M.B., Fender, J.G., & Wartella, E. (2010). Word learning from baby videos. Archives of Pediatrics & Adolescent Medicine, 164(5) 432–437. 10.1001/ARCHPEDIATRICS.2010.24 20194251

[ref84] Richert, R.A., Robb, M.B., & Smith, E.I. (2011). Media as social partners: The social nature of young children’s learning from screen media. Child Development, 82(1), 82–95. 10.1111/j.1467-8624.2010.01542.x 21291430

[ref85] Royal College of Paediatrics and Child Health (2019). The health impacts of screen time: A guide for clinicians and parents. Retrieved from https://www.rcpch.ac.uk/sites/default/files/2018-12/rcpch_screen_time_guide_-_final.pdf

[ref86] Scarf, D., & Hinten, A.E. (2018). Television format and children’s executive function. Pediatrics, 141(3). 10.1542/peds.2017-2674 29467278

[ref87] Sharkins, K.A., Newton, A.B., Albaiz, N.E., & Ernest, J.M. (2015). Preschool children’s exposure to media, technology, and screen time: Perspectives of caregivers from three early childcare settings. Early Childhood Education Journal, 44(5), 437–444. 10.1007/s10643-015-0732-3

[ref88] Shin, E., Choi, K., Resor, J., & Smith, C.L. (2021). Why do parents use screen media with toddlers? The role of child temperament and parenting stress in early screen use. Infant Behavior and Development, 64, 101595. 10.1016/j.infbeh.2021.101595 34153781

[ref89] Smirnova, E.O., Matushkina, N.Y., & Smirnova, S.Y. (2018). Virtual’naia real’nost’ v rannem i doshkol’nom vozraste [Virtual reality in early and preschool childhood]. Psikhologicheskaia nauka i obrazovanie [Psychological Science and Education] 23(3), 42–53. 10.17759/pse.2018230304

[ref90] Soldatova, G.U., & Vishneva, A.E. (2019). Osobennosti razvitiia kognitivnoi sfery u detei s raznoi on-lain aktivnovnost’iu: est’ li zolotaia seredina? [Features of the development of the cognitive sphere in children with different online activities: Is there a golden mean?] Konsul’tativnaia psikhologiia i psikhoterapiia [Counseling Psychology and Psychotherapy], 27(3), 97–118. 10.17759/cpp.2019270307

[ref91] Suggate, S.P., & Martzog, P. (2020). Screen-time influences children’s mental imagery performance. Developmental Science, 23(6) 1–13. 10.1111/desc.12978 32353916

[ref92] Sundararajan , N. , Adesope, O. , & Cavagnetto, A. (2018). The process of collaborative concept mapping in kindergarten and the effect on critical thinking skills. Journal of STEM Education, 19(1) 5–13.

[ref93] Supanitayanon, S., Trairatvorakul, P., & Chonchaiya, W. (2020). Screen media exposure in the first 2 years of life and preschool cognitive development: A longitudinal study. Pediatric Research, 88(6), 894–902. 10.1038/s41390-020-0831-8 32170192

[ref94] Tsai, T.-H., Tseng, K.C., & Chang, Y.-S. (2017). Testing the usability of smartphone surface gestures on different sizes of smartphones by different age groups of users. Computers in Human Behavior, 75, 103–116. 10.1016/j.chb.2017.05.013

[ref95] Twenge, J.M. (2019). iGen: Why today’s super-connected kids are growing up less rebellious, more tolerant, less happy and completely unprepared for adulthood and what that means for the rest of us. Atria.

[ref96] Uġraş, M. , Zengin, E. , Papadakis, S. , & Kalogiannakis, M. (2023). Early childhood learning losses during COVID-19: Systematic review. Sustainability, 15(7), 6199. 10.3390/su15076199

[ref97] Vaiopoulou, J., Papadakis, S., Sifaki, E., Kalogiannakis, M., & Stamovlasis, D. (2022). Classification and evaluation of educational apps for early childhood: Security matters. Education and Information Technologies, 28(3), 2547–2578. 10.1007/s10639-022-11289-w 36035974 PMC9398896

[ref98] Vedechkina, M., & Borgonovi, F. (2021). A review of evidence on the role of digital technology in shaping attention and cognitive control in children. PsyArXiv. 10.31234/osf.io/vjsuw PMC7943608 33716873

[ref99] Veraksa, A.N., Bukhalenkova, D.A., Chichinina, E.A., & Almazova, O.V. (2020). Osobennosti ispol’zovaniia tsifrovykh ustroistv sovremennymi doshkol’nikami [Digital devices use by 6–7 year-old children]. Sotsiologicheskie issledovaniia [Sociological Research], 6, 82–92. 10.31857/S013216250009455-3

[ref100] Veraksa, N.E., Bukhalenkova, D.A., Veraksa, A.N., & Chichinina, E.A. (2022). Vzaimosviaz’ ispol’zovaniia tsifrovykh ustroistv i razvitiia reguliatornykh funktsii u doshkol’nikov [The relationship between the use of digital devices and the development of regulatory functions in preschool children]. Psikhologicheskii zhurnal [Psychological Journal], 43(1), 51–59. 10.31857/S020595920018769-1

[ref101] Walsh, J.J., Barnes, J.D., Tremblay, M.S., & Chaput, J.-P. (2020). Associations between duration and type of electronic screen use and cognition in US children. Computers in Human Behavior, 108, 106312. 10.1016/j.chb.2020.106312

[ref102] Wan, M.W., Fitch-Bunce, C., Heron, K., & Lester, E. (2021). Infant screen media usage and social-emotional functioning. Infant Behavior and Development, 62, 101509. 10.1016/j.infbeh.2020.101509 33249357

[ref103] Warburton, W., & Highfield, K. (2017). Children and technology in a smart device world. In R. Grace, K. Hodge, & C. McMahon (Eds.), Children, families and communities (pp. 195–221). Oxford University Press.

[ref104] Wolf, M. (2021). Prust i kal’mar: neirobiologiia chteniia [Proust and the squid: The neurobiology of reading]. KoLibri.

[ref105] Woodward, J., Shaw, A., Luc, A., Craig, B., Das, J., Hall, P., … & Anthony, L. (2016). Characterizing how interface complexity affects children’s touchscreen interactions. Proceedings of the 2016 CHI Conference on Human Factors in Computing Systems. 10.1145/2858036.2858200

[ref106] World Health Organization. (2019). Guidelines on physical activity, sedentary behaviour and sleep for children under 5 years of age. 31091057

[ref107] Zhang, N., Yang, S., & Jia, P. (2022) Cultivating resilience during the COVID-19 pandemic: A socioecological perspective. Annual Review of Psychology, 73(1), 575–598. 10.1146/annurevpsych-030221-031857. 34579547

[ref108] Zhu, R., Fang, H., Chen, M., Hu, X., Cao, Y., Yang, F., & Xia, K. (2020). Screen time and sleep disorder in preschool children: Identifying the safe threshold in a digital world. Public Health, 186, 204–210. 10.1016/j.puhe.2020.07.028 32861085

[ref109] Ziemer, C.J., Snyder, M. (2016). A picture you can handle: Infants treat touch-screen images more like photographs than objects. Frontiers in Psychology, 7. 10.3389/fpsyg.2016.01253 PMC4992727 27597837

